# The Origin of Mucosal Immunity: Lessons from the Holobiont *Hydra*

**DOI:** 10.1128/mBio.01184-16

**Published:** 2016-11-01

**Authors:** Katja Schröder, Thomas C. G. Bosch

**Affiliations:** Zoological Institute and Interdisciplinary Research Center Kiel Life Science, Christian-Albrechts-University, Kiel, Am Botanischen Garten, Kiel, Germany

## Abstract

Historically, mucosal immunity—i.e., the portion of the immune system that protects an organism’s various mucous membranes from invasion by potentially pathogenic microbes—has been studied in single-cell epithelia in the gastrointestinal and upper respiratory tracts of vertebrates. Phylogenetically, mucosal surfaces appeared for the first time about 560 million years ago in members of the phylum Cnidaria. There are remarkable similarities and shared functions of mucosal immunity in vertebrates and innate immunity in cnidarians, such as *Hydra* species. Here, we propose a common origin for both systems and review observations that indicate that the ultimately simple holobiont *Hydra* provides both a new perspective on the relationship between bacteria and animal cells and a new prism for viewing the emergence and evolution of epithelial tissue-based innate immunity. In addition, recent breakthroughs in our understanding of immune responses in *Hydra* polyps reared under defined short-term gnotobiotic conditions open up the potential of *Hydra* as an animal research model for the study of common mucosal disorders.

## INTRODUCTION

The mucosal immune system is responsible for interfacing with the outside world and modulates an organism’s immune response to microbes approaching the mucosal surfaces at various parts of the body (1). At birth, the neonate’s mucosal immune system is relatively undeveloped, but the colonization of intestinal microbiota accelerates its development (2). Immune tolerance to maintain homeostasis is a hallmark of the mucosal immune system (3). This delicate homeostasis is achieved through an elaborate cross talk between the epithelium and components of the innate and adaptive immune systems, as well as the microbiota. Malfunctioning of this interaction in genetically predisposed individuals is thought to account to a large extent for inflammatory bowel diseases (IBD), such as Crohn’s disease and ulcerative colitis (4).

The microbiota is a central component of mucosal immunity. Strikingly, the trillions of bacteria inhabiting the mucosal surfaces, particularly of the digestive tract, do not induce pathological inflammatory responses or high-titer serum antibody responses (5). Studies in germ-free mice uncovered underdeveloped lymphoid tissues, defective T and B cell function, and low numbers of circulating CD4^+^ T cells and antibody production, all of which can be restored by colonizing mice with microorganisms (6). Similar findings have been described using germ-free zebrafish, which lack specific aspects of gut epithelium differentiation and proliferation and show altered gut motility, all of which can be reversed by the introduction of intestinal microbiota (7, 8). Although lacking adaptive immunity, invertebrates such as *Drosophila* have developed sophisticated regulatory mechanisms to tolerate commensal and mutualistic bacteria in the gut while allowing effective immune responses to clear pathogens (9–11). Thus, the intestinal microbiota and the host mucosal immune system need to be seen as an ecological unit consisting of interacting and exchangeable components, in which the microbiota shapes the immune system and the immune system influences the microbiota composition.

In the past, studies of animal*-*microbe interactions have primarily focused on the detection and killing of pathogens. However, over the last 2 decades, symbiotic host-microbe interactions have become a rapidly advancing research field strongly driven by the emerging awareness that several human diseases result from a shift in the composition of the microbiota (dysbiosis) rather than from invasion by a single pathogen (12, 13). Historically, animal-microbe symbioses have been extensively studied in invertebrates, usually focusing on binary associations involving a single host and a single symbiont. These associations are often mutualistic, coevolved, and remarkably specific, with the host being able to select for the correct symbiotic partners and stably maintain them over ecological and evolutionary time scales (14). In contrast to intracellular symbiosis, such as several arthropod-*Wolbachia* associations (15) and the pea aphid*-Buchnera* symbiosis (16, 17), the colonization by extracellular symbionts requires continuous interaction with the host’s mucosal immune system. A commonly used example of extracellular symbiosis is the partnership between the Hawaiian bobtail squid and the luminous bacterium *Vibrio fischeri*, where host-derived mucus provides a surface upon which *V. fischeri* bacteria aggregate prior to colonization of the crypts of the light organ (18). Studying both intracellular and extracellular symbioses has yielded extensive knowledge of the mechanisms involved in symbiont recognition, selection, and transmission (19). However, apart from these well-studied bipartite model systems, many symbioses range in complexity from hundreds to thousands of microbial symbionts, e.g., the human gut (20), the termite hindgut (21), and marine sponges (22). Despite rapidly growing research on consortial symbioses in the last 2 decades, it remains widely unclear how complex microbial communities colonizing mucosal surfaces are dynamically structured and maintained throughout life.

**FIG 1:**
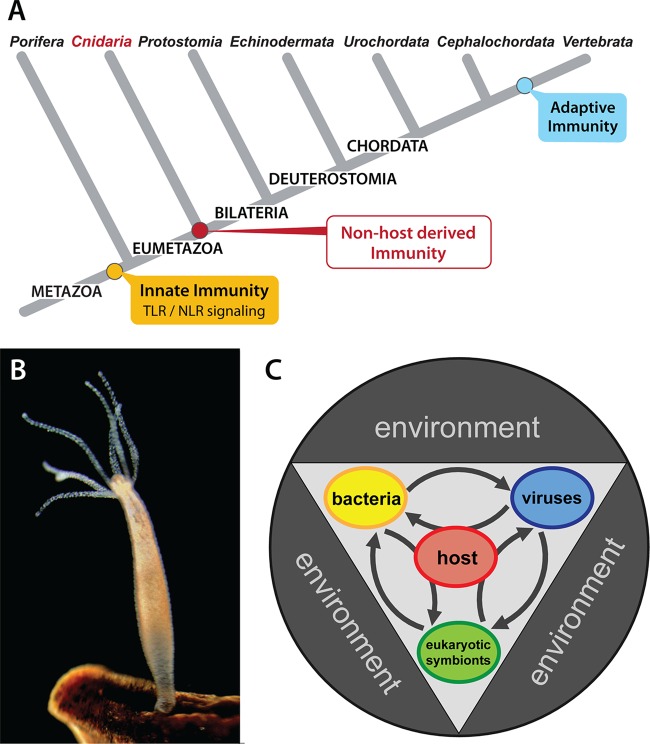
(A) Dendrogram showing evolutionary relationships of selected metazoans. Taxa are arranged in descending order of phylogenetic emergence relative to vertebrates. Divergence times are not to scale, and tree branches are intended only to depict general relationships. TLR, Toll-like receptor; NLR, Nod-like receptor. (B) The freshwater polyp *Hydra vulgaris* attached to substrate. The basal metazoan has been a useful model addressing fundamental questions in immunity and host-microbe interactions in recent years. (C) Multicellular organisms are metaorganisms composed of the macroscopic host and synergistically interdependent bacteria, archaea, viruses, and eukaryotic species, including fungi and algal symbionts (modified from reference 74 with permission of the publisher).

The origin and development of mucosal surfaces represent a major evolutionary step that supported metazoan life (23). Phylogenetically, mucosal surfaces appeared for the first time in members of the Cnidaria (Fig. 1A), eumetazoan animals with a radially symmetrical, sac-like body plan. Can early-emerging metazoans therefore help us to understand basic concepts that may be involved in mucosal immunity? Cnidarians, such as the freshwater polyp *Hydra*, are diploblastic animals consisting of an ectodermal and an endodermal epithelium (Fig. 1B). While both layers are separated by an extracellular matrix (mesoglea), a true mesoderm is missing. In both layers, epitheliomuscular cells whose bodies form part of the epithelium but whose bases extend to form muscle fibers are multifunctional, having both secretory and phagocytic activity. Cnidarians are not only among the earliest known phyletic lineages that form natural symbiotic relationships with bacteria and eukaryotes but also possess most of the gene families found in bilaterians and have retained many genes that have been lost in *Drosophila melanogaster* and *Caenorhabditis elegans* (24, 25). For this reason, early-emerging metazoans like *Hydra* allow us to gain insights into the very early evolution of biological modules that may be involved in mucosal immunity.

## MICROBE-EPITHELIAL INTERACTIONS IN *HYDRA*

In the absence of an adaptive immune system, *Hydra* polyps employ an elaborate innate immune system to detect and interact with microbes using their two cell layers as efficient defense barriers (26). Invading microorganisms first have to overcome the physicochemical barrier represented by the multilayered glycocalyx that covers the ectodermal epithelium (27, 28). Complex cellular and humoral pathways represent the second arm of *Hydra*’s immunity (29). The cellular mechanisms include phagocytosis, tissue repair and regeneration, and apoptotic reactions. Apart from these cellular mechanisms, *Hydra* polyps possess a broad range of antimicrobial factors, such as antimicrobial peptides (AMPs) and kazal 2-type protease inhibitors (29–33). The humoral factors also include pattern recognition molecules (34) that are frequently based on lectin-carbohydrate interactions designed to recognize highly conserved structures present in many different microorganisms (35). Moreover, stem cell transcription factors, such as forkhead box O (FoxO), were found to be involved in controlling the expression of AMPs (36). Humoral pathways therefore appear to be closely interconnected with the establishment and maintenance of tissue homeostasis by stem cell transcription factors in *Hydra.*

Bacteria are important members of the *Hydra* holobiont, or metaorganism (Fig. 1C). Because *Hydra* polyps of various species have been cultivated for more than 20 years in the laboratory at constant temperature and with identical food, it came as a surprise that individuals of these species differed greatly in their microbiotas (37, 38). The bacterial community composition is specific for any given *Hydra* species, and disturbances or shifts in the microbiota can compromise the health and fitness of the whole animal (39). For example, *Hydra* polyps, when artificially deprived of their specific epithelial microbiota, are prone to lethal infection by the filamentous fungus *Fusarium* (39). Furthermore, the species-specific microbial composition parallels the phylogenetic relationships of the *Hydra* species (37, 38). The microbiota, therefore, reflects an ancestral footprint of evolution, a pattern termed phylosymbiosis (40). This finding strongly indicates that distinct selective pressures are imposed on and within the *Hydra* epithelium and that the host cells actively shape the composition of the colonizing microbiota. But the assembly of host-associated microbial communities depends not only on the genetics of the host, it is also affected by contributions of the environment, including intermicrobial interactions. By profiling the assembly of *Hydra*’s microbiota up to 15 weeks posthatching, we observed distinct and reproducible stages of colonization: high initial variability and the presence of numerous different bacterial species are followed by the transient preponderance of the bacterial species that later dominates the adult microbiota. At the end of the colonization process, there is a drastic decrease of diversity (41). To uncover the principal rules of the microbial assembly process in *Hydra*, a replicator-colonizer approach was applied to model the temporal evolution of an interacting bacterial community in a competitive environment (41, 42). This allowed us to suggest that both frequency-dependent bacteria-bacteria interactions and host factors, such as components of the innate immune system, are shaping the succession of microbial colonization in *Hydra* (41). This observation also indicates that the bacterial community within an animal is not static but constantly changing as part of a microevolutionary process and that the process of bacterial colonization is a complex phenomenon where the system’s dynamics cannot be explained by merely adding the properties of its constituents (41).

Thus, numerous observations in *Hydra* suggest that immune systems may have evolved as much to manage and exploit beneficial microbes as to fend off harmful ones (26, 43). As a result of the finding that interactions between animals and microbes are not specialized occurrences but, rather, are fundamentally important aspects of animal biology and that antimicrobial peptides and other components of the immune system are key factors for allowing the right microbes to settle and to kick the less desirable ones out, the view of the role of the immune system has changed radically in the last decade (43–47).

## *HYDRA*’S GLYCOCALYX AND MUCUS LAYER: BOTH A PHYSICOCHEMICAL BARRIER AND A MICROBIAL HABITAT

A characteristic feature of most animal epithelial cells is a dense carbohydrate-rich layer at the apical cell surface, referred to as the glycocalyx (48). The glycocalyx represents a dense forest of highly diverse and constantly renewed transmembrane glycoproteins, proteoglycans and glycolipids (49). Beside mediation of cell-cell recognition and receptor-ligand interactions, the glycocalyx provides an important barrier function to the plasma membrane, best characterized for the intestinal glycocalyx (50–52). In mucosal tissues, such as the mammalian gastrointestinal tract, the glycocalyx is additionally coated by a mucus gel ranging in thickness from 10 µm in the eye up to 700 µm in the large intestine (53). Mucus gels are not membrane-anchored and represent a flexible coat that can move on top of the glycocalyx. In the intestinal tract, the mucus layer is continually shed by movement of the luminal content, whereas in the respiratory tract, cilia drive its movement.

**FIG 2:**
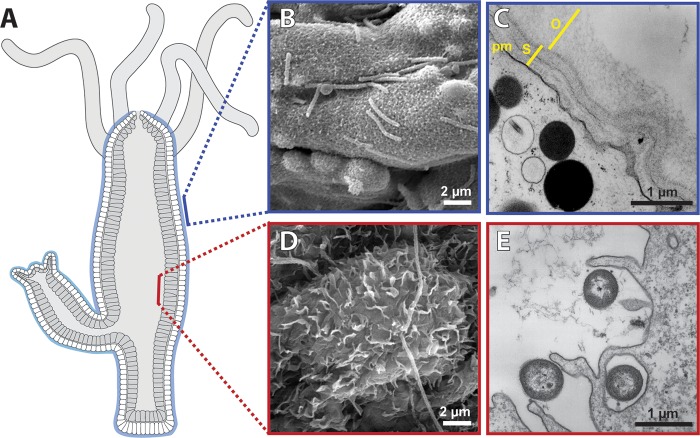
(A) Schematic longitudinal section of a *Hydra* polyp indicating the simple epithelial organization. (B) Scanning electron micrograph (SEM) of the apical surface of the ectodermal epithelium colonized by bacteria. (Modified from reference 39 with permission of the publisher.) (C) Transmission electron micrograph (TEM) of the ectoderm covered by the glycocalyx, which can be subdivided into an inner stratified layer (s) and an outer loose layer (o). pm, plasma membrane. (Modified from reference 39 with permission of the publisher.) (D) SEM of the pseudopod-like structures on the surface of a ciliated endodermal cell. (E) TEM of an endodermal epithelial cell engulfing bacteria. (Modified from reference 29 with permission of the publisher.)

*Hydra*’s tube-like body structure resembles in several aspects the anatomy of the vertebrate intestine, with the endodermal epithelium lining the gastric cavity and the ectodermal epithelium providing a permanent protective barrier to the environment (Fig. 2A). A single layer of ectodermal epithelial cells covered by a multilayered glycocalyx represents a physical barrier toward the environment, whereas a single layer of endodermal epithelial cells separates the body from the content of the gastric cavity. Although once simply considered a physical barrier, it is becoming increasingly evident that *Hydra*’s epithelium is a crucial regulator of microbial homeostasis (29, 30).

The interaction between bacteria and epithelium may differ in the ectoderm and endoderm because of morphological differences. Whereas *Hydra*’s ectodermal epithelium is protected from direct contact with bacteria by the glycocalyx (Fig. 2B and C), the endodermal epithelium does not possess a comparable structure (Fig. 2D). Nevertheless, the endoderm regularly faces various kinds of microbes that are ingested together with the polyp’s prey. Endodermal epithelial cells, therefore, not only contribute to digestion and uptake of food but also phagocytose bacteria present in the gastric cavity (Fig. 2E). *Hydra*’s endoderm appears to be well equipped against bacterial invaders and complements the lack of a physical barrier by producing vast amounts of AMPs belonging to the hydramacin (30), periculin (29, 31), and arminin (32) peptide families. In addition, zymogen gland cells that are found interspersed in the endodermal epithelium supply kazal 2-type serine protease inhibitors possessing bactericidal activity (33). These humoral and cellular defense mechanisms seem to prevent bacterial colonization of the endodermal epithelium lining the gastric cavity. Supporting this view, despite intense examination by different microscopy tools (scanning electron microscopy [SEM], transmission electron microscopy [TEM], and confocal laser scanning microscopy [CLSM]) including various fixation and preparation techniques, so far we did not find evidence for bacterial populations stably colonizing the endodermal epithelial surface lining *Hydra*’s gastric cavity (K. Schröder and T. C. G. Bosch, personal observation).

*Hydra*’s glycocalyx extends up to 1.5 µm from the cell surface and is composed of at least five morphologically distinguishable layers (Fig. 2C) (27, 28). All layers of *Hydra*’s glycocalyx, as well as the large secretory vesicles underneath the apical membrane, were shown to be highly periodic acid-Schiff stain (PAS) reactive, indicating a high carbohydrate content (28). Although the structure was initially termed glycocalyx, the characteristic feature of being membrane bound does not account for all of its layers. The outer layer (o), which accounts for more than 50% of the glycocalyx, is made of a loose meshwork that can be removed completely by a hypertonic salt wash. In contrast, the four inner layers (s) display a dense composition and are firmly attached to the epithelial surface (28). Therefore, we propose that the outer layer of *Hydra*’s glycocalyx has mucuslike properties rather than being a part of the membrane-anchored glycocalyx. The mucuslike layer of *Hydra*’s glycocalyx provides the habitat for the symbiotic bacterial community (39). Bacteria were never observed reaching the dense inner layers of the glycocalyx or even the ectodermal cell membrane. Thus, *Hydra*’s glycocalyx has two functionally distinct compartments: an outer mucuslike layer that forms the habitat for the microbiota and an inner stratified layer that is most likely membrane bound and acts as a physicochemical barrier. Strikingly, a similar observation was made in the mammalian colon. An inner firmly adherent layer with stratified organization was devoid of bacteria, whereas the outer loose layer appeared to be colonized by symbionts (54, 55). The separation of the mucus layer into a barrier and a habitat for the microbiota might, therefore, represent a conserved principle.

We previously demonstrated that after exposure of *Hydra* polyps to filtrates of adherent grown *Pseudomonas aeruginosa* bacteria, ectodermal cells form numerous blebs at the apical surface and secretory vesicles underneath the cell membrane strongly increase (29). Thus, similar to more complex organisms, such as vertebrates, *Hydra*’s epithelial cells respond to microbial stimuli by cytoskeletal rearrangement and increased secretory activity. However, even in nonstimulated polyps, we find some PAS-reactive vesicles permanently at the apical surface, suggesting that *Hydra*’s glycocalyx is constantly renewed and rebuilt. Indeed, after removal of the outer layer by a salt wash, the polyp is able to completely restore the outer layer in less than 6 h (K. Schröder, unpublished data). Thus, similar to the intestinal mucus in mammals (56), constant renewal of the glycocalyx most likely allows *Hydra*’s rapid adjustments to a constantly changing environment.

## DEEP-TIME EVOLUTION OF MUCOSAL IMMUNITY

**FIG 3:**
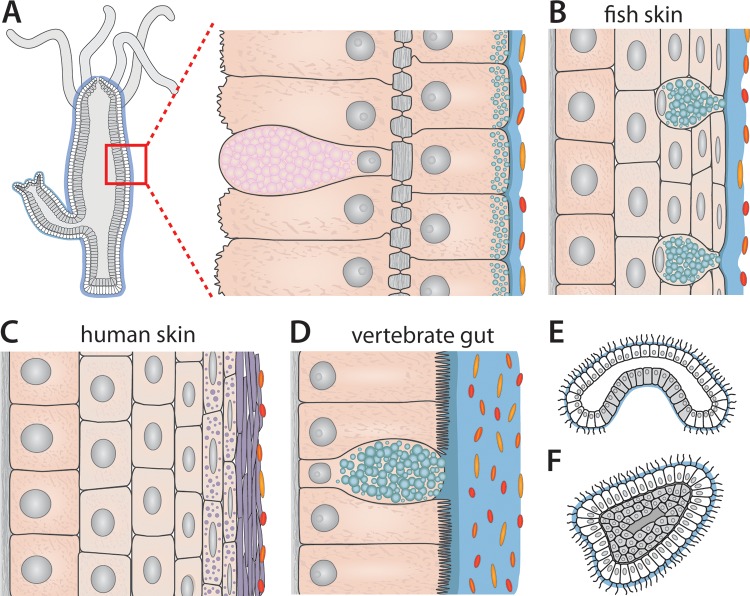
Schematic representation of different types of mucosal epithelia across metazoan evolution. (A) Longitudinal section of a *Hydra* polyp displaying the simple body plan formed by an epithelial bilayer. The ectoderm is covered by the glycocalyx, which provides the habitat for bacteria. (B) Fish skin is a mucosal surface that is formed by a stratified epithelium harboring mucus-producing cells and lacking keratinization. (C) The human epidermis as an example of the keratinized, stratified epithelia of terrestrial vertebrates. Instead of a mucus gel, flattened remnants of dead cells form the physical barrier of the skin. This cornified envelope efficiently prevents the loss of vital fluids, which displays an essential adaptation to terrestrial life. (D) The monolayered epithelium of the vertebrate gut is covered by mucus that forms two distinct layers: an inner layer devoid of bacteria and an outer layer that is heavily colonized by symbionts. (E) Pelagic, planktotrophic gastraea as a hypothesized metazoan ancestor. (F) Larva representing the planktotrophic stages in life cycles of early-emerging marine invertebrates.

All multicellular organisms require an effective immune system either to suppress hostile microbes, to maintain a beneficial microbiota, or at least, to tolerate epithelial colonization. Launching an immune response comes at a cost, consuming extra resources and energy. Between efficiency and cost, metazoans are driven to evolve immune mechanisms and strategies to achieve a dynamic balance (57). Toward this goal, as summarized above, early-emerging metazoans have used their single-cell epithelia to develop an effective innate immune system to detect and control microbial colonization (Fig. 3A). Over the past 560 million years of vertebrate evolution, additional and complex mechanisms of systemic and mucosal defenses have evolved. These include mucosal surfaces with their mucosa-associated lymphoid tissues, as well as cutaneous tissues (58–60). How the evolutionary transition between the cutaneous surfaces and mucosal surfaces was realized remains elusive. Early vertebrates, such as fish, originated in aquatic environments, and therefore, their skin behaves as a mucosal surface that harbors abundant mucus-producing cells, lacks keratinization, and is built from living epithelial cells that are in direct contact with the water medium (Fig. 3B) (61). For successful radiation of vertebrates in terrestrial environments, the ancient ancestors of modern tetrapods had to overcome the problem of desiccation. Important structural changes of the skin, namely, loss of mucus production accompanied by the acquisition of keratinized cell layers, reduced the loss of water (Fig. 3C). In addition, respiratory surfaces evolved within the body cavity and gills were replaced by lungs. Amphibians are the first vertebrates that successfully exploited terrestrial environments, while remaining closely tied to water or moist microhabitats for reproduction. Most amphibians experience rapid desiccation in dry environments. With the emergence of reptiles, the outer mucosal surface of the skin became completely replaced by an effective skin barrier, reducing that rapid and extensive water loss in reptiles. In addition to the cutaneous tissue, mucosal surfaces in the vertebrate gut (Fig. 3D) at the same time harbor high numbers of bacteria and also prevent the luminal microbiota from penetrating the intestinal mucosa and from spreading systemically.

The evolutionary origin of the mucosal surfaces remains enigmatic. Information from the fossil record, from the occurrence of epithelial-like cells, and from genetics give no direct knowledge about the ancestral mucosal surface. Insight, however, may be obtained by considering evolutionary developmental aspects. Haeckel’s famous gastraea theory (62) proposed that all metazoans have evolved from a pelagic, planktotrophic ancestor called gastraea; benthic adult stages consequentially appeared later in the life cycle of certain lineages (Fig. 3E). In 2013, a seminal paper by Claus Nielsen (63) provided strong support for this theory, suggesting that (i) the ancestor of the eumetazoans was a holopelagic, planktotrophic gastraea (Fig. 3E) lacking adult forms, (ii) benthic adult stages were added secondarily, and (iii) planktotrophic stages in life cycles are represented by larvae (Fig. 3F). This also implies that adult stages of cnidarians were later additions to the life cycle (Fig. 3A). Thus, in accordance with Nielsen’s view on animal evolution (63), we propose that the origin of mucosal surfaces predates the emergence of Cnidaria and that mucosal surfaces first were developed by the various larval types of early emerging marine invertebrates.

## WHAT CAN WE LEARN FROM *HYDRA*?

Defining the individual host-microbe cross talk in a given holobiont (Fig. 1C) is a challenging but necessary step on the path to understanding the function of the associations as a whole. Untangling the complex interactions requires simple animal models with only a few specific bacterial species. Such models can function as living test tubes and may be the key to dissect fundamental principles that underlie all host-microbe interactions. Here, we have introduced the cnidarian *Hydra* as such a nontraditional model to characterize innate immune responses at epithelial barriers (29), tissue homeostasis (36), and host-microbe interactions (37–39). *Hydra* is developmentally well characterized and amenable to genetic manipulation (64), and polyps are easily propagated as clonal lines, and can be maintained for several weeks in the absence of microbes (germ-free). Since in addition the genome (65) and the epithelial organization are remarkably similar to those of vertebrate mucosal surfaces, these animals offer unique insights into the biology of epithelial barriers.

**FIG 4:**
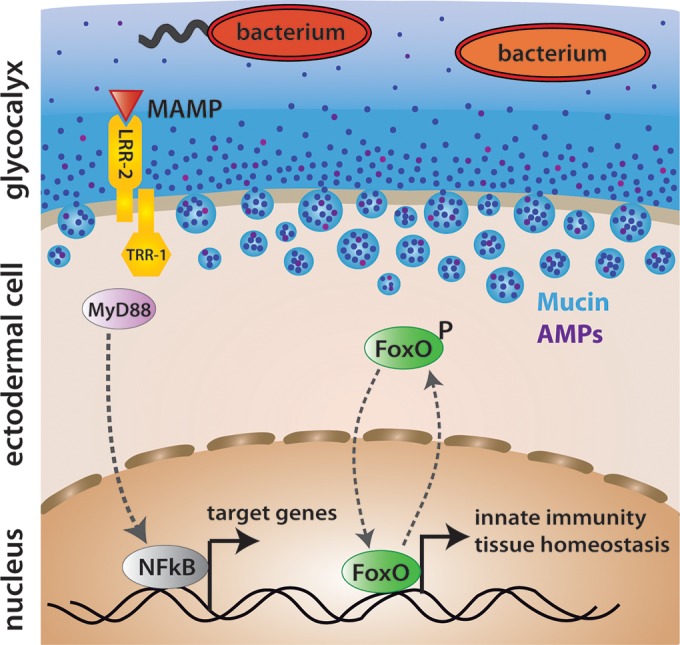
In the holobiont *Hydra*, there is a direct link between stem cell proliferation, innate immunity, and microbiota composition. Innate immune recognition in *Hydra* is mediated by Toll-like receptor (TLR) signaling. Upon activation, the receptor recruits primary adaptor molecules, such as MyD88, to engage downstream signaling pathways. Tissue homeostasis and immunity are linked by stem cell transcription factors, such as FoxO. Target genes of FoxO include antimicrobial peptides (AMPs). Ectodermal epithelial cells also secrete mucins to establish the mucus and glycocalyx layer.

For example, it was surprising to discover that in *Hydra*, there is a direct link between stem cell proliferation, innate immunity, and microbiota composition. As schematically shown in Fig. 4, the detection of microbes is achieved by pattern recognition receptors (PRRs) that detect conserved molecular structures known as microbe-associated molecular patterns (MAMPs) (Fig. 4). RNA interference (RNAi) knockdown experiments with *Hydra* Toll-like receptors (TLRs) showed a drastic reduction of antimicrobial activity in the knockdown tissue compared to that in the wild-type tissue, which makes it apparent that antimicrobial activity relies directly on the activation of the TLR cascade (29). Upon MAMP detection, TLRs recruit an adaptor protein, MyD88 (Fig. 4), resulting in the transcriptional activation of downstream immune response genes. Engagement of these receptors leads to a fast induction of protective programs, e.g., the induction of antimicrobial peptides or the elimination of the infected cell by means of apoptosis (66). Interestingly, silencing of stem cell transcription factor FoxO activity not only affects developmental and differentiation genes but also causes changes in the expression of antimicrobial peptides, which often reflect the immune status of *Hydra* (36). The link between FoxO and components of the innate immune system indicates that in *Hydra*, developmental pathways represented by the stem cell transcription factor FoxO are tightly coupled to innate immunity. Overall, the unexpected link between FoxO and innate immunity has shed at least some light on the age-old problem of how developmental pathways are linked to components of the innate immune system.

Another important insight was that in *Hydra*, non-host-derived immunity plays a major role in protecting mucosal surfaces and disturbances of the microbial community can compromise the health of the whole animal (39). While wild-type *Hydra* very rarely show signs of fungal infection, removing the epithelial microbiota results in lethal infection by the filamentous fungus *Fusarium*. Most importantly, recolonization of gnotobiotic polyps with monoassociations of bacterial colonizers failed to provide complete protection. Fungal resistance is only achieved by restoring the complex microbiota. Multiple members of the microbiota act synergistically to confer resistance against the pathogenic fungus, indicating that functional diversity within the microbiota likely is central to pathogen clearance from the epithelium. These results highlight the importance of additive and synergistic interactions within the microbial community to provide full pathogen resistance. The increasing recognition of the concept of critical colonization and the appreciation of microbe-derived factors affecting mucosal homeostasis may aid the development of more effective methods of treating mucosal disorders.

The final example concerns the role of viruses in a holobiont. Viruses are the most abundant and diverse biological component on the planet, found in any environment where cellular life exists (67–69). But despite a growing appreciation that all types of organisms, including bacteria, plants, fungi and animals, are hosts to certain viruses, little is known about the viruses that reside in and on mucosal surfaces (70, 71). We recently could demonstrate that, in *Hydra*, not only the bacterial microbiota but also the viral communities are specific for each host species tested (72). Strikingly, a large portion of viruses associated with *Hydra* turned out to be bacteriophages, accounting for 38 to 63% of the virus-sequencing hits. Thereby, the classes of predicted bacteriophage hosts reflect the species-specific bacterial communities of *Hydra*. This observation suggests a potential role for phages in regulating the bacterial microbiota in the *Hydra* holobiont. Indeed, this hypothesis seems to be corroborated by a recent study analyzing the *in vitro* bacterial population dynamics of two symbionts (*Curvibacter* sp. and *Duganella* sp.) that synergistically protect the *Hydra* host from fungal infection (39, 73). The observed frequency-dependent, nonlinear growth rates indicate that the interactions among these two bacteria in coculture are beyond the simple case of direct pairwise interactions. One possible explanation capturing the complexity of the system includes the effect of phage infection. Our hypothesis is that a bacteriophage infecting *Curvibacter* sp. at a population equilibrium level may switch its bacterial host to *Duganella* sp. and, thus, induce an outbreak event, which reduces the growth rate by increased mortality.

## CONCLUSIONS

In multicellular animals ranging from *Hydra* to humans, the highly glycosylated and hydrated glycocalyx and mucus layer are the first physical, chemical, and biological barrier to infection and are also a habitat for bacteria. The mucus composition is complex and includes numerous factors secreted by epithelial cells to discriminate between pathogens and commensal or mutualistic microorganisms. Extraordinary recent progress in sequencing technologies and the ability to culture simple but genetically accessible model organisms for some time under germ-free conditions are revealing details of host-microbe interactions that undermine prior concepts and highlight the value of an evolutionary perspective. However, in spite of these insights, we still do not know the factors involved in microbial colonization of mucosal surfaces. We also have not been able to coherently integrate the accumulated abundance of information into a truly mechanistic understanding of host-microbe interactions, in particular how a complex microbiota interacts as a spatially and temporally dynamic network on host mucosal surfaces. What is urgently needed is the integration of information across numerous organisms, from early-emerging metazoans to vertebrates, including humans, on multiple levels of organization, portraying the ecological and genetic interaction networks of entire systems and moving away from a linear cause-and-effect perspective. Disturbed host-microbial interactions and related inflammatory signaling play an important role in the etiology of several chronic inflammatory disorders affecting barrier organs such as the intestine. Functional studies are crucial to elucidate the causal mechanisms by which microbes affect host fitness and how human genetic variation impacts the microbiota to identify novel treatments for mucosal disorders. As shown here, nontraditional model systems, such as *Hydra*, may serve as informative experimental tools in rethinking paradigms in medical research.
